# Comparative Genomics of Potato Common Scab-Causing *Streptomyces* spp. Displaying Varying Virulence

**DOI:** 10.3389/fmicb.2021.716522

**Published:** 2021-08-03

**Authors:** Cindy Hudec, Adrien Biessy, Amy Novinscak, Renée St-Onge, Simon Lamarre, Jochen Blom, Martin Filion

**Affiliations:** ^1^Department of Biology, Université de Moncton, Moncton, NB, Canada; ^2^Saint-Jean-sur-Richelieu Research and Development Centre, Agriculture and Agri-Food Canada, Saint-Jean-sur-Richelieu, QC, Canada; ^3^Agassiz Research and Development Centre, Agriculture and Agri-Food Canada, Agassiz, BC, Canada; ^4^Bioinformatics and Systems Biology, Justus-Liebig-Universität Giessen, Giessen, Germany

**Keywords:** common scab, *Streptomyces*, genomics, pathogenicity island, virulence, thaxtomin

## Abstract

Common scab of potato causes important economic losses worldwide following the development of necrotic lesions on tubers. In this study, the genomes of 14 prevalent scab-causing *Streptomyces* spp. isolated from Prince Edward Island, one of the most important Canadian potato production areas, were sequenced and annotated. Their phylogenomic affiliation was determined, their pan-genome was characterized, and pathogenic determinants involved in their virulence, ranging from weak to aggressive, were compared. 13 out of 14 strains clustered with *Streptomyces scabiei*, while the last strain clustered with *Streptomyces acidiscabies*. The toxicogenic and colonization genomic regions were compared, and while some atypical gene organizations were observed, no clear correlation with virulence was observed. The production of the phytotoxin thaxtomin A was also quantified and again, contrary to previous reports in the literature, no clear correlation was found between the amount of thaxtomin A secreted, and the virulence observed. Although no significant differences were observed when comparing the presence/absence of the main virulence factors among the strains of *S. scabiei*, a distinct profile was observed for *S. acidiscabies*. Several mutations predicted to affect the functionality of some virulence factors were identified, including one in the *bldA* gene that correlates with the absence of thaxtomin A production despite the presence of the corresponding biosynthetic gene cluster in *S. scabiei* LBUM 1485. These novel findings obtained using a large number of scab-causing *Streptomyces* strains are challenging some assumptions made so far on *Streptomyces’* virulence and suggest that other factors, yet to be characterized, are also key contributors.

## Introduction

Potato plants are subjected to various stresses, including diseases caused by numerous bacterial, oomycete and fungal plant pathogens, affecting yield and market values (Stevenson et al., 2001). Common scab (CS) of potato is highly prevalent in Prince Edward Island (PEI), which is one of the most important potato-producing Canadian provinces (Statistics Canada, 2021). CS causes necrotic lesions on the tuber surface, which affect potato transformation and commercialization, resulting in economic losses estimated between 15.3 and 17.3 million dollars per year in Canada (Hill and Lazarovits, 2005). This worldwide disease is caused by a few closely related *Streptomyces* species (Braun et al., 2017). Among the dozen *Streptomyces* species causing CS reported so far, the best characterized are *Streptomyces scabiei*, *Streptomyces acidiscabies*, and *Streptomyces turgidiscabies* (Loria et al., 2006).

Advances in next-generation sequencing technologies have resulted in an upsurge of bacterial genome sequences available. However, so far only few plant pathogenic *Streptomyces* spp. genomes have been sequenced (18 *S. scabiei*; ten *S. acidiscabies*, and two *S. turgidiscabies*, NCBI May 2021). Although these genomic sequences have provided new opportunities to perform comparative genomic studies, additional genomes will be required to perform large-scale analyses. Comparisons between pathogenic and saprophyte *Streptomyces* spp. have revealed numerous genes and gene clusters that contribute to pathogenicity, virulence and/or plant-microbe interactions (i.e., pathogenome) (Bignell et al., 2010). Moreover, functional analyses of some of these genetic determinants have confirmed their implication in the development of CS symptoms (Bignell et al., 2010).

The main pathogenicity determinant in scab-causing *Streptomyces* spp. has been identified as a cyclic dipeptide (2,5-diketopiperazine) phytotoxin called thaxtomin A, which inhibits cellulose biosynthesis on actively growing tubers (Lawrence et al., 1990; King et al., 1991; Bignell et al., 2010). A positive correlation between the severity of the symptoms observed (virulence) and thaxtomin A production has also been reported (Kinkel et al., 1998). Genes involved in thaxtomin A biosynthesis, consisting of a cluster of six genes: *txtA, txtB, txtC, txtD, txtE*, and *txtH* (Bignell et al., 2014b), are localized in a *cis-*mobilizable element (CIME) in a toxicogenic region (TR1, 20 kb) (Zhang and Loria, 2017). This region is highly conserved among thaxtomin-producing *Streptomyces* spp. and is separated from a TR2 region by an internal attachment site (*att*) (Chapleau et al., 2016). TR2 (157 kb) contains putative integrative and conjugative elements (ICE) required for mobilization and is only conserved among *S. scabiei* strains (Zhang et al., 2016).

Biosynthesis of thaxtomin A is under the strict transcriptional control of the TxtR protein, a member of the AraC/XylS family, which promotes the transcription of *txtA*, *txtB*, *txtC*, and *txtH* (Johnson et al., 2007; Joshi et al., 2007). More specifically, cellobiose and cellotriose, which derive from cellulose present in the plant cell wall, are the main inducers of thaxtomin A production (Johnson et al., 2007). The ability of pathogenic *Streptomyces* to participate in the degradation of plant polymers like cellulose or suberin, have been demonstrated through the secretion of glycosyl hydrolases and esterases such as suberinase and cutinase (Komeil et al., 2013; Padilla-Reynaud et al., 2015; Huguet-Tapia et al., 2016). These two oligosaccharides are specifically transported inside *S. scabiei* by the CebEFG/MsiK transmembrane transporter (Jourdan et al., 2016). Once internalized, they interact with CebR, a transcriptional repressor of the thaxtomin A biosynthetic gene cluster. In the absence of these oligosaccharides, CebR binds to DNA via two CebR-binding sites (*cbs*; *cbs*^*txtB*^ and *cbs*^*txtA–R*^) localized inside *txtB* and between the *txtA* and *txtR* genes, repressing the expression of these *txt* genes. When cellobiose and cellotriose interact with CebR, the repression is lifted and thaxtomin A production begins (Francis et al., 2015). This production is also under the control of several *bld* genes (*bldA*, *bldC*, *bldD*, *bldG*, and *bldH*) principally known to participate in the morphological development and/or the production of antibiotics by *Streptomyces* spp. (McCormick and Flärdh, 2012; Bignell et al., 2014a).

In addition to thaxtomin A, and notably due to the existence of pathogenic *Streptomyces* spp. that do not produce this toxin (Park et al., 2003; Wanner, 2004; Cao et al., 2012; Fyans et al., 2016; Lapaz et al., 2017), others phytotoxins and/or secreted proteins produced by pathogenic *Streptomyces* spp. have been described or are suspected to play a role in the development and/or the severity of the disease. Among them, the production of coronafacoyl (N-coronafacoyl-L-isoleucine; CFA-Ile) (Fyans et al., 2015; Bignell et al., 2018) and concanamycin A and B (Natsume et al., 1996, 1998; Fyans et al., 2016) have been reported in *S. scabiei.* Their respective biosynthetic gene clusters have never been found in other *Streptomyces* species causing CS. As for thaxtomin A, some *bld* genes have also been identified as transcriptional regulators of its production (Bignell et al., 2014a). Even if their role in CS development remains unclear, it has been demonstrated that they are non-essential pathogenicity factors but still significantly contribute to the severity of the disease as virulence factors (VFs). On potato tuber tissues, CFA-Ile has been shown to increase necrosis and pitting (Cheng et al., 2019), and concanamycin A and B, which have a synergic effect with thaxtomin A, have also been shown to contribute to the formation of pitted lesions (Natsume et al., 2017).

Recent studies have revealed the production of additional phytotoxins by *Streptomyces* spp. also impacting lesion types and/or disease severity. *Streptomyces* sp. strain GK18 isolated from a scab lesion in Iran was shown to produce borrelidin, a macrolide causing deep and black holes on potato tuber slices (Cao et al., 2012). A strain of *Streptomyces niveiscabiei* (strain ST1015) isolated in Uruguay was shown to produce desmethylmensacarcin, a polyketide more aggressive than thaxtomin A that also causes deep necrotic lesions on potato tubers (Lapaz et al., 2019). Several phytopathogenic *Streptomyces* strains isolated in Japan produce macrolide FD-891, which induces necrosis on potato tuber slices (Natsume et al., 2005). Natsume et al. (2018), have also isolated a *S. turgidiscabies* strain in Sweden producing fridamycin E, which reduces or inhibits the sprouting of potato microtubers (Natsume et al., 2018).

In addition to the phytotoxins secreted by scab-causing *Streptomyces* spp., these pathogens produce phytohormones which facilitate the infection of potato plants, notably by altering the plant hormonal signaling pathways. Kers et al. (2004) identified a gene cluster in *S. turgidiscabies* highly similar to the plant fasciation (*fas*) operon present in *Rhodococcus fascians*. In this species, the *fas* operon is required for pathogenicity and allows the biosynthesis of cytokinin (CK), a plant growth hormone (Eason et al., 1996). CK production by *S. turgidiscabies* and its involvement in the pathogenicity was then confirmed by Joshi and Loria (2007). The production of auxin (indole-3-acetic acid, IAA) in *S. scabiei* was also demonstrated (Bignell et al., 2010) and its contribution to virulence was observed (Hsu, 2010; Legault et al., 2011). A homolog of a gene encoding an ethylene-forming enzyme (*efe*) that catalyzes ethylene production (Bignell et al., 2010), which could be involved in the colonization of the pathogen *in planta* (Weingart et al., 2001), was also described in *S. scabiei*.

Finally, in addition to these phytotoxins and phytohormones, scab causing *Streptomyces* spp. are also able to secrete proteins (effectors or extracellular enzymes) which contribute to virulence. For example, in *S. scabiei*, scabin belonging to the mono-ADP-ribosyltransferase family, was identified as a VF (Lyons et al., 2016). The *nec1* and *tomA* genes, which encode, respectively, a necrotic protein and a putative tomatinase probably involved in the suppression of plant defense responses have also been reported in some pathogenic *Streptomyces* spp. as VFs (Loria et al., 2006). Both of these genes are located in a chromosomal region of 105 kb conserved among *Streptomyces* species, the colonization region (CR) (Lerat et al., 2009; Zhang et al., 2016). In *S. turgidiscabies*, this region and the TR region belong to a mobile pathogenicity island (PAI) that can be horizontally transferred from pathogenic to saprophytic *Streptomyces* species, resulting in the emergence of new pathogenic phenotypes (Kers et al., 2004; Zhang et al., 2016). Despite its polyphyletic origin, *S. scabiei* appears to be the oldest common scab-causing pathogen that has participated to the emergence of others pathogenic species such as *S. acidiscabies* and *S. turgidiscabies*, through lateral gene transfer (Chapleau et al., 2016; Huguet-Tapia et al., 2016).

In a recent study performed in our laboratory, a collection of close to 300 prevalent scab-causing *Streptomyces* strains were isolated from PEI, Canada and grouped into fourteen distinct genetic groups of *Streptomyces* spp. (Hudec et al., 2021). Thirteen of them were taxonomically affiliated with *S. scabiei*, while the last one was affiliated with *S. acidiscabies.* Moreover, virulence assays showed strong differences in virulence, ranging them from weak (producing less than 5% of CS coverage on tubers) to aggressive strains (>50% tuber coverage) (Hudec et al., 2021). In order to elucidate why such unexpected variability in virulence was observed, the genomes of these 14 scab-causing genetic groups of *Streptomyces* spp. were sequenced and compared. First (i), pan-genome description and phylogenetic relationships between the fourteen strains were investigated to study putative links existing between virulence and general strain affiliations. Secondly, (ii) toxicogenic and colonization regions containing several pathogenic determinants were compared. Thirdly, (iii) thaxtomin A production was quantified using standardized assays to determine its implication in virulence, and finally (iv) genes involved directly or indirectly in the biosynthesis and regulation of thaxtomin A production and others known virulence determinants were compared to identify potential genetic mutations altering thaxtomin A production and/or virulence. The analyses performed in this study provide up-to-date information on the genomic diversity of pathogenic *Streptomyces* spp. found under commercial potato production conditions in Eastern Canada as well as its implication in virulence. A better characterization of the determinants contributing to the virulence in pathogenic *Streptomyces* strains is required for the development of adapted management tools under field conditions.

## Materials and Methods

### Bacterial Strains, Culture Conditions and Conservation

The 14 pathogenic *Streptomyces* strains used in this study were previously isolated from potato tubers (cv. Prospect) harvested in PEI, Canada (Hudec et al., 2021). Originally named *Streptomyces* spp. G01–G14 (genetic groups 1–14), these strains were renamed *Streptomyces scabiei* LBUM 1475, LBUM 1477, LBUM 1487, LBUM 1480, LBUM 1485, LBUM 1482, LBUM 1486, LBUM 1484, LBUM 1483, LBUM 1488, LBUM 1481, LBUM 1478, LBUM 1479, and *Streptomyces acidiscabies* LBUM 1476, respectively. The strains were grown in the dark at 28°C under continuous shaking (120 rpm) for 4 days using 100 ml of tryptic soya broth (TSB, BD Biosciences, Franklin, NJ, United States), and were conserved at −80°C in TSB supplemented with 50% glycerol (vol/vol).

### Genome Sequencing, Assembly and Annotation

Genomic DNA was extracted using the *DNeasy UltraClean Microbial Kit* (Qiagen, Mississauga, ON, Canada) as previously described (Hudec et al., 2021) and purified using Agencourt AMPure XP beads (Beckman Coulter, Mississauga, ON, Canada) according to the manufacturer’s instructions.

Genomes were sequenced using Single Molecule, Real-Time sequencing technology on a PacBio RS II sequencer (Pacific Biosciences, Menlo Park, CA, United States) and assembled using the HGAP pipeline (Chin et al., 2013) at the McGill University and Genome Quebec Innovation Centre located in Montreal, QC, Canada.

The genome sequences obtained in this study were annotated using the *Prokaryotic Genome Annotation Pipeline* (*PGAP*) (Tatusova et al., 2016) and were deposited in DDBJ/ENA/GenBank under the accession numbers provided in Table 1. The versions described in this paper are the first versions.

**TABLE 1:** General genomic information on the 14 scab-causing *Streptomyces* spp. under study.

Species	Strains	Genome coverage	Contig no.	Contig N50 (Mb)	Genome size (Mb)	G + C content (%)	CDSs no. (total)	Accession numbers
*S. scabiei*	LBUM 1475	159x	1	9.74	9.74	71.6	8455	CP043958
*S. acidiscabies*	LBUM 1476	140x	13	10.35	10.94	70.6	9751	VWSX00000000
*S. scabiei*	LBUM 1477	148x	5	9.57	9.77	71.6	8528	VWSW00000000
*S. scabiei*	LBUM 1478	63x	116	6.03	10.15	71.3	9004	VWSV00000000
*S. scabiei*	LBUM 1479	78x	3	9.62	10.19	71.5	8911	VWSU00000000
*S. scabiei*	LBUM 1480	153x	1	9.98	9.98	71.5	8654	CP043957
*S. scabiei*	LBUM 1481	112x	5	9.52	9.87	71.5	8569	VWST0000000
*S. scabiei*	LBUM 1482	167x	1	9.78	9.78	71.6	8518	CP043956
*S. scabiei*	LBUM 1483	148x	3	9.68	9.82	71.5	8548	VWSS00000000
*S. scabiei*	LBUM 1484	100x	2	9.59	9.63	71.6	8373	VWSR00000000
*S. scabiei*	LBUM 1485	58x	58	6.76	9.87	71.5	8616	VWSQ00000000
*S. scabiei*	LBUM 1486	127x	3	9.88	10.05	71.5	8803	VWSP00000000
*S. scabiei*	LBUM 1487	147x	2	9.79	9.97	71.5	8695	VWSO00000000
*S. scabiei*	LBUM 1488	169x	2	9.94	9.98	71.5	8630	VWSN00000000

### Thaxtomin A Quantification Using LC-MS

*Streptomyces* strains were first cultured in TSB as previously described. Standardization of the bacterial quantities, culture conditions in oat bran broth media and collection of the supernatants were performed as described by Jiang et al. (2018). Solid phase extraction cartridges (Bond-Elut-C18, 100 mg, 1 ml, Agilent Inc., Mississauga, ON, Canada) were used to extract thaxtomin A. These columns were first preactivated with 1 ml of 100% methanol (MeOH) and washed with an equal volume of water. Then, 1 ml of sample was passed through the preactivated cartridge followed by 1 ml of water and 1 ml of 25% MeOH in water. Thaxtomin A was then eluted using 0.25 ml of 100% MeOH. The eluate was combined with one volume of water with 0.1% formic acid (solvent A) immediately before being analyzed using liquid chromatography with mass spectrometry detection (LC-MS). The chromatographic separation was performed using a reverse phase HPLC column (Synergi Hydro-RP, 100 mm × 2 mm, Phenomenex Inc., Torrance, CA, United States). The LC conditions included an injection volume of 5 μl into a column at 37°C eluted with a linear gradient of 0–100% solvent B (methanol:acetonitrile 50:50) over 10 min at a flow rate of 0.5 ml/min. The column was further cleaned with 100% solvent B for 3 min and then reequilibrated with solvent A for 10 s. Isolated thaxtomin A was detected in time-of-flight MS. The ion source was a dual electrospray (ESI) operated on positive mode. The MS conditions were as follows: gas temperature, 325°C; capillary voltage, 3500 V; fragmentor, 175 V; and skimmer, 65 V. One MS spectra per second was acquired under a mass range of m/z 50–1,700. Under these conditions thaxtomin A was eluted at 7.39 min with m/z 439.1612. The concentrations of thaxtomin A were extrapolated from a standard curve of pure thaxtomin A from 0 to 2 μg/ml (Sigma-Aldrich Canada Co., Oakville, ON, Canada). In cases where the sample concentration was higher than 2 μg/ml, the samples were further diluted using 0.1% formic acid in water. The chromatographic system used was an Agilent 1100 and the MS system was an Agilent 6230 (Agilent Inc.). Data acquisition was performed using the MassHunter Version B.08.00 software. The experiment set-up consisted of three independent biological replicates. Statistical analyses were performed using RStudio version 1.2.5001 (Boston, MA, United States). The R function “kruskal” with a Benjamini-Hochberg (BH) correction, from the “Agricolae” package version 1.2-8 was used (*p* < 0.05) to compare the thaxtomin A produced among the different strains (De Mendiburu, 2019). A correlation between the amount of thaxtomin A produced *in vitro* and the common scab coverage observed *in planta* as published in a previous study (Hudec et al., 2021) (i.e., virulence) was performed using the Kendall method (De Mendiburu, 2019).

### Taxonomy and Phylogeny

To determine the species affiliation of the 14 strains under study, two analyses were performed using the type strains *S. scabiei* NRRL B-16523^*T*^ and *S. acidiscabies* NRRL B-16524^*T*^. The first one was performed using *in silico* DNA-DNA hybridization (*is*DDH) with Genome-to-Genome Distance Calculator – Formula 2 (GGDC 2.1) on the web-based DSMZ service available at http://ggdc.dsmz.de (Meier-Kolthoff et al., 2013). A cut-off of 70% for species affiliation was used. The second one was based on the Average Nucleotide Identity (ANI) and was calculated using the algorithm developed by Goris et al. (2007), with a 95% cut-off for species boundary (Richter and Rosselló-Móra, 2009). We report here the averages of the reciprocal comparisons obtained on http://enve-omics.ce.gatech.edu/ani/ (Rodriguez-R and Konstantinidis, 2016). Moreover, a phylogenomic tree of the 14 *Streptomyces* strains under study, as well as eight additional well characterized pathogenic *Streptomyces* species was built using EDGAR 3.0 (Dieckmann et al., 2021). *Streptomyces griseolus* NRRL B-2925^*T*^, a non-pathogenic *Streptomyces* species was used as an outgroup. The robustness of the inferred tree was evaluated with a bootstrap resampling method with 1000 replicates.

### Pan-Genome

Coding DNA sequences (CDSs) from each genome were compared against each other to identify orthologous CDSs that are (i) present in every strain (core-genome), (ii) species- or group-specific, or (iii) unique to a given strain (singletons) using EDGAR 3.0. Orthologs were identified using bidirectional best BLAST hits using a Score Ratio Value of 0.31 as the orthology cutoff.

In order to provide information on the specific functions of the CDSs belonging to the pan-genome of these 14 strains, the protein sequences were annotated against the Clusters of Orthologous Groups database (COGs; Tatusov et al., 2000) by BLASTP search (*e*-value < 1 × 10^–3^) with eggNOG-mapper v2 databases (Huerta-Cepas et al., 2017) and against the Virulence Factor Database (VFDB; *e*-value < 10^–5^) (Liu et al., 2019).

### Analysis of the Genetic Determinants Involved in Pathogenicity and Virulence

Based on the literature, known gene sequences related to *Streptomyces*’ pathogenicity and virulence (Supplementary Table 3) were retrieved from the genomes of the 14 *Streptomyces* strains using BLASTn. The toxicogenic and colonization regions were also identified. Multiple sequence alignments (MAFFT) of these genes and gene regions were performed (Katoh and Standley, 2013) and Neighbor-Joining consensus trees based on the Jukes-Cantor distance were constructed (Saitou and Nei, 1987). To evaluate the robustness of the inferred trees, the bootstrap resampling method was used with 1000 replicates. These analyses were performed on Geneious Prime 2019.2.3 (Biomatters, Ltd., Auckland, New Zealand).

All nucleotide sequences of the 13 *S. scabiei* were then compared with those of the reference strain *S. scabiei* 87-22 (NC_013929.1), while sequences of *S. acidiscabies* LBUM 1476 was compared with the reference strain *S. acidiscabies* 84-104 (NZ_AHBF00000000.1). Sequences were then translated to determine the impact of mutations on protein sequences. The effect of amino acid substitutions were then analyzed using Protein Variation Effect Analyzer (PROVEAN)^1^ (Choi and Chan, 2015). Variants with a score equal to or below −2.5 were considered “deleterious,” contrary to variants with a score above −2.5 which were considered as “neutral.” The predicted secondary structures of tRNA were determined using tRNAscan-SE^2^ (Lowe and Chan, 2016).

## Results

### General Characteristics of the Genome Sequences

The genomes of 14 common scab-causing *Streptomyces* strains isolated from diseased tubers sampled in Canadian commercial potato production fields were sequenced, assembled, and annotated. Genomic features, including chromosome size, G + C% content, CDSs and contig numbers, are presented in Table 1. Briefly, the chromosome sequence sizes vary between 9.63 and 10.94 Mbp (average 9.98) with a total number of CDSs ranging between 8373 and 9751 (average 8718) and a G + C% fluctuating between 70.6 and 71.6% (average 71.4). Each genome contains six rRNA operons (containing 5S, 16S, and 23S rRNA) and between 70 and 75 tRNA genes.

### Phylogeny and Taxonomy

*In silico* DNA-DNA hybridization and ANI calculations were performed to estimate genome similarity and taxonomical affiliation between the 14 *Streptomyces* strains under study. All *Streptomyces* strains except strain LBUM 1476 belong to the *S. scabiei* species with an average GGDC and ANI values of 95.68 and 99.56%, respectively. LBUM 1476 was instead associated with *S. acidiscabies* with a GGDC of 99.2% and ANI of 99.97%. For each of the 14 *Streptomyces* strains, the GGDC and ANI values yield similar results for species discrimination (Supplementary Table 2).

**FIGURE 1:**
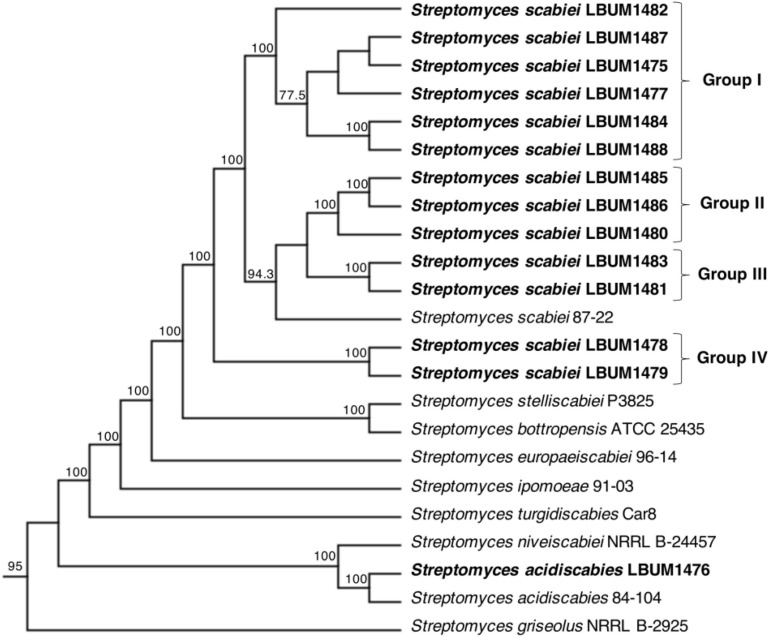
Neighbor-joining phylogeny tree of 14 *Streptomyces* strains used in this study (bold) and eight phytopathogenic *Streptomyces* spp. reference strains. *S. griseolus* NRRL B-2925 was used as an outgroup. The Neighbor-Joining tree is based on the alignment of the core-genomes of these organisms (corresponding to 1577 CDSs per genome). Four *S. scabiei* groups were distinguished. Only bootstrap values higher than 60% are displayed. Branches are presented as a cladogram for improved clarity.

A phylogenomic tree of the 14 *Streptomyces* strains, alongside eight additional pathogenic *Streptomyces* spp. and an outgroup was constructed (Figure 1). This tree was based on the core-genome of these 23 strains, that corresponds to 1577 CDSs. This analysis showed consistent results with earlier observations using either ANI or GGDC values.

Four groups of *S. scabiei* could been distinguished according to an arbitrary threshold of GGDC > 98% and ANI > 99.80%. These groups were supported in the tree by 100% bootstrap values. The *S. scabiei* group I is the largest group comprising *S. scabiei* LBUM 1475, LBUM 1477, LBUM 1482, LBUM 1484, LBUM 1487, and LBUM 1488. All strains belonging to this group produce the melanin pigment contrary to those belonging to group II that include *S. scabiei* LBUM 1480, LBUM 1485, and LBUM 1486 (Hudec et al., 2021). Groups III and IV which also produced melanin, are the smallest groups and contain *S. scabiei* LBUM 1481/LBUM 1483 and LBUM 1478/LBUM 1479, respectively. Group IV is was the most distinct when compared to the other three groups.

### Analysis of a Potential Link Between the Phylogenetic Groups and Virulence

To determine if a link exists between the phylogenetic groups identified and the virulence observed in a previous study (Hudec et al., 2021), we compared the *S. scabiei* group affiliation with the severity of symptoms observed (Table 2). Globally, strains belonging to the *S. scabiei* groups I and II display weak to intermediate virulence contrary to group IV, which contains intermediate to aggressive strains. Group III presents a higher virulence variability (Table 2). These results suggest the existence of distinct virulence factors or differential production of these factors between, and in some cases among the different *S. scabiei* groups.

**TABLE 2:** *In silico* analysis of the pathogenic determinants involved in pathogenicity and/or virulence of scab-causing *Streptomyces* spp., and their relative virulence and thaxtomin A production.

			Phytotoxins						Phytohormones			Secreted proteins				Virulence^a^*	Thaxtomin A production^a^
Phylogenetic group	Species	Strain	Thaxtomin A	*C*oronafacoyl	Conca	Desmethylm	Borrelidin	FD-891	Cytokinins	Auxin	Ethylene	Nec1	TomA	Scabin	Suberinase		
					namycin A	ensacarcin											
I	*S. scabiei*	LBUM 1482	+	+	+	−	−	−	−	+	+	+	+	+	+	+	++
I	*S. scabiei*	LBUM 1487	+	+	+	−	−	−	−	+	+	+	+	+	+	+	+
I	*S. scabiei*	LBUM 1477	+	+	+	−	−	−	−	+	+	+	+	+	+	+	++
I	*S. scabiei*	LBUM 1475	+	+	+	−	−	−	−	+	+	+	+	+	+	++	+
I	*S. scabiei*	LBUM 1484	+	+	+	−	−	−	−	+	+	+	+	+	+	++	++
I	*S. scabiei*	LBUM 1488	+	+	+	−	−	−	−	+	+	+	+	+	+	++	++
II	*S. scabiei*	LBUM 1485	+	+	+	−	−	−	−	+	+	+	+	+	+	+	−
II	*S. scabiei*	LBUM 1486	+	+	+	−	−	−	−	+	+	+	+	+	+	+	++
II	*S. scabiei*	LBUM 1480	+	+	+	−	−	−	−	+	+	+	+	+	+	++	+++
III	*S. scabiei*	LBUM 1481	+	+	+	−	−	−	−	+	+	+	+	+	+	+	+++
III	*S. scabiei*	LBUM 1483	+	+	+	−	−	−	−	+	+	+	+	+	+	+++	+++
IV	*S. scabiei*	LBUM 1479	+	+	+	−	−	−	−	+	+	+	+	+	+	++	+++
IV	*S. scabiei*	LBUM 1478	+	+	+	−	−	−	−	+	+	+	+	+	+	+++	+++
–	*S. acidis cabies*	LBUM 1476	+	−	−	+	−	−	−	+	+	+	+	+	−	+++	+

^*a*^+indicates weak, ++ intermediate, and +++ strong virulence or thaxtomin A production.

*The virulence data were retrieved from a previous *in planta* virulence assays performed in our laboratory (Hudec et al., 2021).

### Characterization of the Pan-Genome

**FIGURE 2:**
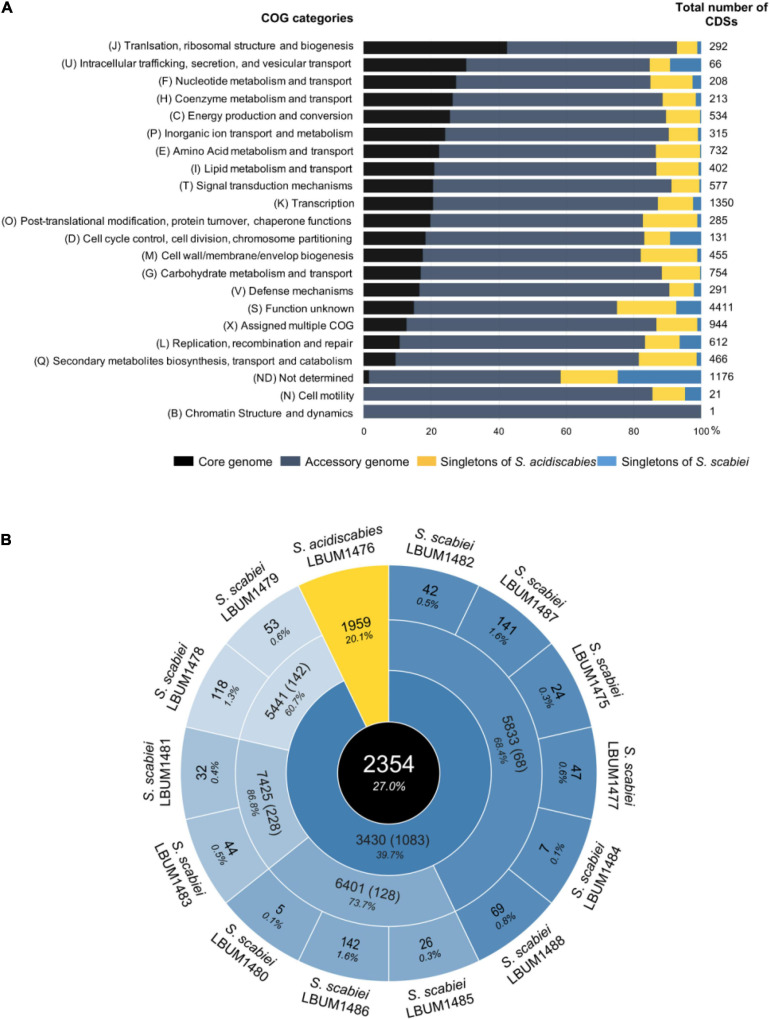
Distribution of the orthologous CDSs from the pan-genome of the 14 scab-causing *Streptomyces* strains under study based on the COG categories **(A)** and their numbers belonging to the core-genome, group-specific, and strain specific genomes **(B)**. In the histogram, bars are sorted by the proportion of CDS associated with the core-genome, the accessory genome and singletons (in *S. acidiscabies* and *S. scabiei*) in each functional category **(A)**. The numbers presented in the circles from inside to outside: The black circle shows the core-genome of the 14 strains used in this study. The second circle shows the core-genome of each species – i.e., the 13 *S. scabies* (blue) and the only *S. acidiscabies* (yellow). The third circle compares the different *Streptomyces scabiei* core-genomes. Finally, the fourth and outermost circle represents the singletons (i.e., the CDSs that are strain-specific). The numbers in brackets indicate the number of CDSs that are species or group-specific (i.e., CDSs present in every strains from a species or a group but absent in all other strains). The percentages indicate the proportion of core genes (or singletons) compared to the average number of CDSs in the relevant set of strains (e.g., the core-genome of 2302 CDSs found for the 14 strains from *Streptomyces* represent, in average, 26.4% of the total number of CDSs for each strain from this set) **(B)**.

To further characterize and compare the functional intra- and inter-species diversity, the pan-genome, which represents the entire gene repertory of the 14 *Streptomyces* under study, was analyzed (Figure 2). A total of 14,236 CDSs was recorded, of which almost half were poorly characterized (categories S, ND, and X). 23% of the characterized CDSs were involved in metabolism (C, E, F, G, H, P, and Q categories), 19% in information storage processing (B, I, J, K, and L categories), and 13% in cellular processes and signaling (including the others COG categories). Moreover, among the 14,236 CDSs identified, 129 were found in VFDB (data not shown).

The *S. acidiscabies* isolate and the 13 *S. scabiei* strains shared 2354 genes (i.e., core-genome) which principally encode essential function such as transcription (K; with 11.7%) and amino acid metabolism and transport (E; 6.9%). Only a small or no portion of the core-genome was associated with cell motility (N; 0%), defense mechanisms (V; 2%), and secondary metabolites biosynthesis (Q; 1.9%). A higher prevalence of these gene categories was instead found to be group- or strain- specific. Moreover, 67% (85) of the VFs identified in this study were shared among the 14 strains (data not shown). The accessory genome which corresponds to all the genes present in two or more strains but not all contains a total of 9173 genes. *S. acidiscabies* and *S. scabiei* contain 1959 (including 15 VFs) and 1083 (21 VFs) species-specific genes, respectively. Only 6 VFs were found to be shared between at least two of the 14 strains, but were not group or species-specific (data not shown). These VFs were (with few exceptions) mostly present in groups II and IV and were involved in transcriptional regulation (3), stress responses (2), and the last one was a transposase.

The *S. scabiei* pan-genome (11,069 genes) included 3430 genes shared among the four *S. scabiei* groups. Among each *S. scabiei* group, more than 60% of genes were shared by all strains belonging to this group, displaying a high intra-group conservation. Each of these groups presents several group-specific genes (from 68 in group I to 228 in group III), mainly involved in replication, recombination and DNA repair categories (L). The N, V, and Q categories potentially involved in pathogenesis and virulence displayed no differences in proportion between the four groups. Moreover, none of the VFs identified were *S. scabiei* group-specific (data not shown). Finally, a total of 750 unique genes were found among the 13 *S. scabiei* strains, and again, no match was found against the VFDB (data not shown).

### Comparative Analysis of the Pathogenicity Island

**FIGURE 3:**
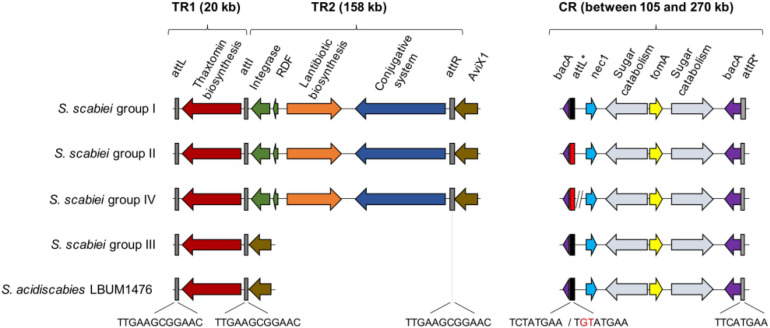
Comparative analysis of the organization of the pathogenicity island between the fourteen scab-causing *Streptomyces* spp. used in this study. Each color corresponds to a gene or a cluster of genes homologous between the fourteen strains.

To better understand the distinct patterns of virulence observed in a previous study using the same 14 *Streptomyces* strains (Hudec et al., 2021), the pathogenicity islands (PAI) containing several pathogenic determinants were compared. Conservation of the PAI organization among each *S. scabiei* group was observed (Figure 3). More specifically, TR1 which contains the entire biosynthetic gene cluster for thaxtomin A production was highly conserved with 97.3% pairwise identity. However, TR2 was not found in *S. scabiei* group III and *S. acidiscabies* LBUM 1476.

The CR region displayed 66.7% pairwise identity, and two distinct organizations were observed: the classical one with 105 kb delimited by the *attL*^∗^ and *att*R^∗^ sites, containing *nec1*, *tomA* and several genes involved in the sugar catabolism; and a second one found only in *S. scabiei* group IV, containing a large insert of 165 kb (Figure 3). This insert was a concatenation of five fragments separated by diverse transposases. These fragments were found in all the strains belonging to *S. scabiei* group II, outside the CR region. Moreover, two distinct palindromic *att*L^∗^ sites were distinguished. The TGTATGAA sequence was found in *S. scabiei* groups II and IV contrary to the TCTATGAA sequence found in the other *S. scabiei* groups and in *S. acidiscabies* LBUM 1476 (Figure 3). In the 14 strains, a degenerated version of *intSt* identified in *S. turgidiscabies* was found. MAFFT alignment of these sequences presented 94.3% of pairwise identity (data not shown).

### Production of Thaxtomin A

**FIGURE 4:**
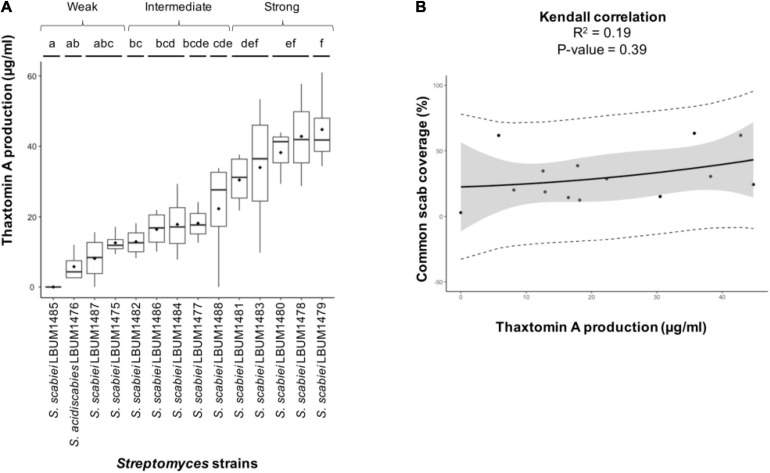
Quantification of thaxtomin A production by the fourteen scab-causing strains of *Streptomyces* used in this study **(A)** and its correlation with the severity of common scab coverage symptoms observed on potato tubers in a previous study (Hudec et al., 2021) **(B)**. Lowercase letters above the bars refer to significant differences (*P* < 0.05).

Thaxtomin A is considered to be the main pathogenic determinant in scab-causing *Streptomyces*. In this context, its production by the 14 *Streptomyces* strains under study was quantified using a standardized assay to determine if differential production between the strains can explain their difference in virulence. Thus, thaxtomin A production and secretion was quantify by LC-MS. Results are presented in Figure 4. On average, *S. acidiscabies* LBUM 1476 produced 5.8 μg of thaxtomin A per ml of supernatant. Except for strain LBUM 1485, which did not produce any detectable amount of thaxtomin A, *S. scabiei* strains produced larger amounts of thaxtomin A than *S. acidiscabies* LBUM 1476. Indeed, concentrations ranging between 8.1 (*S. scabiei* LBUM 1487) and 44.8 μg/ml (LBUM 1479) are reported here (Figure 4A). Surprisingly, no correlation between the amount of thaxtomin A produced *in vitro* and the severity of symptoms observed *in planta* was found (Figure 4B; Hudec et al., 2021).

Moreover, similar production within group III (on average 32.3 μg/ml) and IV (43.8 μg/ml) was observed. A higher heterogeneity was observed for the *S. scabiei* strains belonging to groups I and II, but, overall, these groups produced lower amounts of thaxtomin A than groups III and IV.

### Analysis of the Genetic Background Involved in the Production of Thaxtomin A

The 14 *Streptomyces* strains under study produced different amounts of thaxtomin A. In order to elucidate the genetics differences involved in this differential production, the entire biosynthetic gene cluster involved in thaxtomin A production (including *txtA*, *txtB*, *txtC*, *txtD*, *txtE*, and *txtH* genes) was compared. A MAFFT alignment of the concatenate CDSs was performed and showed 99.9% pairwise identity and 99.3% identical sites (data not shown). Conversion into protein sequences identified a total of 37 amino acid (AA) substitutions (Supplementary Table 3). Only one of these AA substitutions (a tyrosine at the position 166 substituted by a histidine; Y166H) was predicted to have an impact on the functionality of the nitric oxide synthase encoded by the *txtD* gene in *S. scabiei* LBUM 1475, LBUM 1481, and LBUM 1483 (Supplementary Table 3).

In addition, all the known genes involved in the regulation of thaxtomin A production were also compared (*txtR*, *bldC*, *bldD*, *bldG*, *bldH, CebE, CebF, CebG, MsiK*, and *CebR*). None of the 12 AA substitutions found were predicted to impact the biological function of the corresponding proteins. Moreover, no nucleotide mutation was found on CebR binding sites among the strains. Finally, one *S. scabiei* strain (LBUM 1485) presented a mutation in *bldA* which is a tRNA Leu-tRNA^*UUA*^. In this case, its impact on the secondary structure was analyzed. This mutation was not found on any conservative loops (the anticodon, the D- and TΨC loop). The base substitution (G6A) observed was on the acceptor stem and is predicted to disrupt the pairing with the corresponding nucleotide (C77) (Supplementary Figure 1).

### Comparative Presence/Absence and Protein Sequence Variations in Virulence Determinants Among the 14 *Streptomyces* spp.

Although thaxtomin A is considered as the main virulence determinant in *Streptomyces* spp. causing CS, others phytotoxins, phytohormones and secreted proteins have been characterized as playing a role in the development and/or the severity of the disease. The 13 *S. scabiei* displayed the same virulence genetic background including the presence of all the genes required for the biosynthesis and regulation of the phytotoxins thaxtomin A, CFA-Ile, and concanamycin A; the phytohormones auxin and ethylene; and the secreted proteins Nec1, TomA, scabin, and suberinase. *S. acidiscabies* LBUM 1476, for its part, presented a distinct pattern of virulence determinants. It does not harbor the genes involved in the biosynthesis of CFA-Ile, concanamycin A and suberinase, but it harbors those involved in desmethylmensacarcin biosynthesis. None of the *Streptomyces* strains under study has the genetic determinants required for the production of borrelidin, FD-891, or cytokinins (Table 2).

Since no distinct genetic virulence pattern seems to explain the varying level of virulence observed for the 14 *Streptomyces* strains, the potential impact of genetic mutations in the genes encoding the biosynthesis and regulation of these virulence determinants was analyzed (Supplementary Table 3). A total of 305 mutations which substitute the corresponding AA of the genes encoding the CFA-Ile, concanamycin A, auxin, ethylene, TomA, and suberinase was observed in *S. scabiei* strains. All of these mutations were group-specific. Indeed, all strains belonging to a given group presented the same mutation. No mutation in the genes encoding mensacarcin, or the Nec1 and scabin proteins was identified. Thus, among the mutations observed, only 35 were predicted to have an impact on the functionality of the corresponding proteins (Table 3). 29 of these were found in the concanamycin biosynthetic gene cluster and none of them were found in the regulatory gene corresponding to SCAB_84101 in *S. scabiei* 87.22. Three mutations were found in the coronatine-like metabolite biosynthetic gene cluster, with one in the *orf1* potentially involved in the regulation of its production. Finally, two mutations were found in the *iaaM* gene (tryptophan monooxygenase enzyme) involved in the indole-3-acetamide (IAM) pathway and one in the gene encoding an ethylene forming enzyme (*efe2*).

**TABLE 3:** Protein mutations encoded by genes involved in the production and/or regulation (bold) of pathogenicity/virulence factors in *Streptomyces* spp. used in this study.

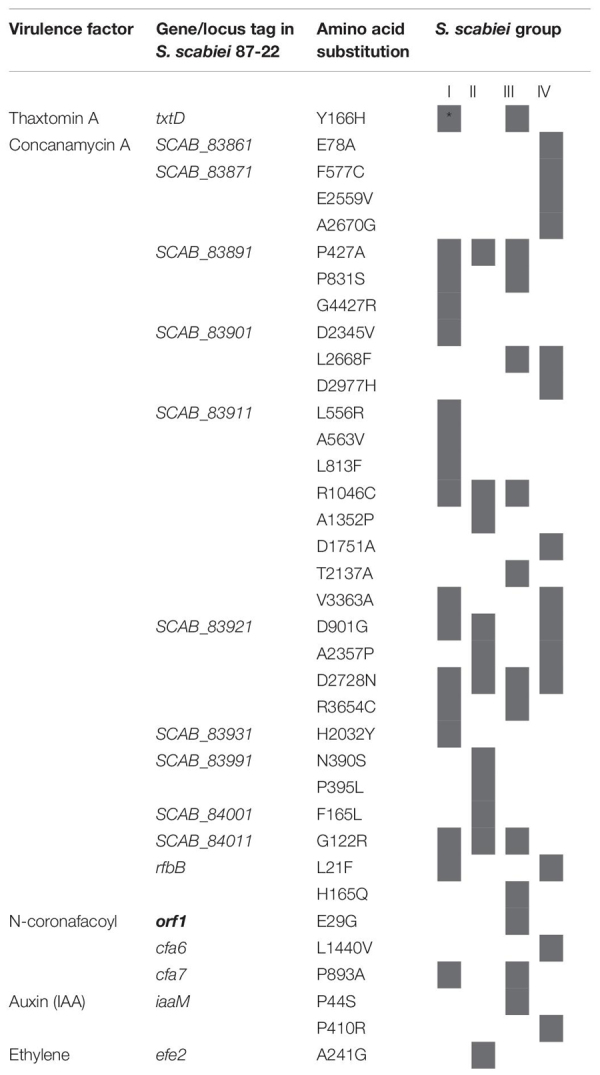

*Sequences were compared to the reference strains *S. scabiei* 87-22 and mutations are indicated in gray.*

*^∗^The mutation was only found in *S. scabiei* LBUM 1475. Bold refers to genes involved in regulation.*

The MAFFT alignment of the concatenated protein sequences showed 99.7% of pairwise identity. Moreover, all virulence determinants seem to have evolved in a group-specific way. Thus, contrary to the core-genome tree (Figure 1), the *S. scabiei* group I seems to be more closely affiliated with group III, then with groups II and IV (Supplementary Figure 2).

## Discussion

In this study, we presented a comprehensive comparative genomic analysis of fourteen scab-causing *Streptomyces* strains (13 *S. scabiei* and one *S. acidiscabies*) with a focus on their PAI and VFs in order to better understand the origin of their distinct virulence patterns observed in a previous study (Hudec et al., 2021).

The pan-genome analyses of these fourteen strains showed that the proportion of the core-genome range from 24.1 to 28.1% in each strain. With the lowest core-genome proportion (24.1%) and the highest number of genes (9751), *S. acidiscabies* LBUM 1476 was the more divergent strain under study. Another study performed on the genome of five *Streptomyces* species had previously identified a core-genome varying between 33 and 45% in each species (Zhou et al., 2012). In our study, with only two distinct species, we would have expected the proportion of common genes to be higher. Thus, this low proportion suggests a high heterogeneity between these fourteen strains, which was not only due to the presence of one *S. acidiscabies* strain. This is confirmed by the proportion of genes shared between the 13 *S. scabiei* strains, corresponding to 39.7% of their gene content. In comparison, another study performed on *Streptomyces* species had shown between 85 and 88% of genes shared among five *Streptomyces pratensis* strains (Doroghazi and Buckley, 2014). It is generally assumed that in a typical bacterial genome, a high proportion of the accessory genome is often associated with horizontal gene transfer (HGT) (Kim et al., 2015; Chater, 2016). Thus, the low proportion of core genes and the high proportion of accessory genes observed in *S. scabiei* species (62.2%) suggests widespread HGT within this species, which may have contributed to the high heterogeneity. It is not surprising since it has already been demonstrated that *Streptomyces* spp. are able to perform HGT within and between species belonging to this genus (Doroghazi and Buckley, 2010). This highlights the existence of a high intra-species heterogeneity inside *S. scabiei*. In pathogenic bacterial strains, the accessory genes are often associated with virulence. Indeed, a loss in some of these genes is usually accompanied by a reduction in virulence (Medini et al., 2005). Here, no correlation between the virulence observed in a previous study and the size of the accessory genome determined in this study was found, suggesting that the varying virulence is more strain-specific than species-specific. Indeed, a study performed on several strains belonging to the *Streptomyces albus* species concluded that each stain harbors at least one strain-specific biosynthetic gene cluster (Seipke, 2015). In this study, a total of 750 *S. scabiei* strain-specific genes were identified. However, none of them was associated with the biosynthesis of any known virulence factor.

Despite this heterogeneity, the 13 *S. scabiei* strains used in this study displayed the same pathogenome, distinct from the one of *S. acidiscabies* LBUM 1476. Thus, despite the existence of common genes involved in the biosynthesis of several VFs, some of them were species-specific. The presence of the biosynthetic gene clusters involved in the biosynthesis of thaxtomin A, Nec1, or TomA in both species was not surprising because these proteins were classically found in *Streptomyces* spp. causing CS (Bignell et al., 2010). Moreover, the absence of the genes coding for the biosynthesis of FD-891, borrelidin, or the *fas* operon involved in the biosynthesis of cytokinins was also expected as these virulence factors have so far only been found in other *Streptomyces* species (notably in *S. turgidiscabies*) (Kers et al., 2004; Natsume et al., 2005, 2018; Cao et al., 2012). The presence of the biosynthetic gene clusters involved in CFA and concanamycin A production exclusively found in *S. scabiei* species was also expected (Natsume et al., 1996, 1998; Fyans et al., 2015, 2016; Bignell et al., 2018). However, until now the production of scabin, auxin and the presence of the gene encoding an ethylene-forming enzyme (*efe*) were only described in *S. scabiei* (Weingart et al., 2001; Bignell et al., 2010; Hsu, 2010; Legault et al., 2011). Here, homologs of the genes involved in their biosynthesis were also found in *S. acidiscabies* LBUM 1476. Moreover, it is also the first time that the genes coding for the biosynthesis of desmethylmensacarcin are found in *S. acidiscabies*. Indeed, among the *Streptomyces* spp. causing CS, this phytotoxin was only previously identified in *S. niveiscabiei* strain ST1015 (Lapaz et al., 2019). Its production by *S. acidiscabies* needs to be further investigated. Finally, among the enzymes which participates to the degradation of plant polymers to enhance thaxtomin A production, suberinase was only found in *S. scabies* (Komeil et al., 2013; Padilla-Reynaud et al., 2015). Its absence in *S. acidiscabies* may explain why this species produced less thaxtomin A than *S. scabiei* under the conditions used in this study.

The sequencing of thirteen *S. scabiei* genomes has allowed us to study in-depth the intra-species diversity. It is, however, not the first time that genetic heterogeneity inside *S. scabiei* is reported (St-Onge et al., 2008; Lapaz et al., 2017). Indeed, genotypic studies using DNA-DNA hybridization have previously distinguished at least three distinct groups within *S. scabiei* (Tashiro et al., 1990; Healy and Lambert, 1991; Paradis et al., 1994). Moreover, these *S. scabiei* groups were able to infect distinct crops as well as distinct potato cultivars with varying virulence (Keinath, 1989; Goyer and Beaulieu, 1997; Wanner, 2006). In our study, the analyses of the core-genome-based phylogeny and *in silico* genome similarity (ANI and *is*DDH) has allowed us to distinguish four distinct *S. scabiei* genomic groups (groups I–IV).

Kers et al. (2004) identified the first PAI (PAISt, 674 Kb) in *Streptomyces turgidiscabies*. Including the *txt* cluster, the *fas* operon, the *nec1* and *tomA* genes, this PAISt was described as a combination of two modules of 105 and 569 Kb delimited by three *att* sites TTCATGAA (*attL* and *attR* at the extremity and *attI* between both modules). This PAI corresponds to an integrative and conjugative element (ICE) which may be entirely excised from the chromosome (by the recombinase IntSt and the putative excisionase XisSt) or partially excised (each individual module) and mobilized into a non-pathogenic strain through conjugation to be integrated into the receiving chromosome at a specific site (Kers et al., 2004; Huguet-Tapia et al., 2011, 2014).

In other *Streptomyces* spp. causing CS, this PAI was separated in two distinct regions by 4.9 Mb: the toxicogenic and the colonization regions, TR and CR, respectively (Lerat et al., 2009). In these species, and notably in *S. scabiei* and *S. acidiscabies*, only remnants of *intSt* and distinct *attL* sequences bordering the CR were found (TGTATGAA for *S. scabiei* and TCTATGAA for *S. acidiscabies*) (Huguet-Tapia et al., 2014). In addition, in *S. scabiei*, the TR region was further divided into two sub-regions, the first one containing the *txt* cluster (TR1), and the second containing an antibiotic biosynthesis cluster, and potential ICE (TR2) (Chapleau et al., 2016). It has been demonstrated that for the strains containing TR2, each of the subregions and the full TR may be excised (Chapleau et al., 2016), but only TR and TR2 can be transferred and integrated specifically in a aviX1 site of a receiving strain (Zhang et al., 2016). Thus, TR1 is considering as a *cis-*mobilizable element (CIME) for which the TR2 (an ICE) is necessary to its mobilization (Zhang and Loria, 2017). Only the receiving strains having acquired the entire TR region develop a pathogenic phenotype and may participated to the emergence of new pathogenic species in an agricultural system (Kers et al., 2004; Zhang et al., 2016).

In our study, the four identified *S. scabiei* groups harbor four distinct organizations of the TR (TR1 and TR2) and CR regions. The TR1 region, which includes all the genes involved in the biosynthesis of thaxtomin A, was conserved among the fourteen *Streptomyces* spp. under study. Huguet-Tapia et al. (2016) previously demonstrated a possible acquisition of this region through lateral gene transfer that resulted in high conservation among *Streptomyces* sp. causing CS. However, contrary to what was previously reported by Zhang et al. (2016), the TR2 region was not conserved among the *S. scabiei* strains under study, since strains from group III do not harbor it. However, this observation supports data published by Chapleau et al. (2016) who identified pathogenic *S. scabiei* strains without a TR2 region. It was previously demonstrated that the TR1 region cannot be transferred to a saprophytic *Streptomyces* strain without the presence of the TR2 region (Zhang and Loria, 2017). Therefore, the absence of the TR2 region supports the assumption that strains belonging to the *S. scabiei* group III cannot contribute to the emergence of new pathogenic *Streptomyces* strains.

The CR region containing the *nec1* and *tomA* genes is also known to be conserved among pathogenic scab-causing *Streptomyces* strains (Zhang et al., 2016). Interestingly, the *S. scabiei* strains belonging to group IV presented a large insert, probably due to several transposition events inside the genome. Indeed, this sequence was found to be a combination of five genomic regions separated by several transposases. These five sequences were also found in the *S. scabiei* strains belonging to group II, suggesting that group IV might have evolved from group II. Moreover, this region is known to be delimited by an imperfect 8 bp palindromic *attL*^∗^ site presenting different nucleotide residues on the second and third base pairs depending on the species. However, in this study, *S. scabiei* strains from groups II to IV presented the *attL*^∗^ sequence identical to the one usually associated with *S. acidiscabies* species. It has already been suggested that the CR region may have been transferred in pathogenic *Streptomyces* spp. genomes via different horizontal gene transfer events than those associated with the TR region (Zhang et al., 2016). In this hypothesis, it would appear that this region may have been mobilized from *S. acidiscabies* to *S. scabiei*, but this will require further investigations.

Despite these four distinct organizations of the TR and CR regions, no correlation between the *S. scabiei* group affiliation of the 13 strains and virulence was found. Moreover, none of the VFs retrieved in this study were group specific. Despite the high heterogeneity found inside *S. scabiei*, no VFs identified at the species, group or strain level can explain the distinct pattern of virulence observed, suggesting the existence of other VFs presently unknown. This varying virulence may also perhaps be explained by the differential production of different VFs.

Indeed, it has already been demonstrated that a positive correlation exists between the amount of thaxtomin A produced and virulence (Kinkel et al., 1998; Loria et al., 2006). Despite the high conservation of the gene cluster involved in the biosynthesis of this phytotoxin, quantification of thaxtomin A produced *in vitro* showed a high variation in the amount produced between the strains under study. The *S. scabiei* strains from groups III to IV, which contain the most virulent strains, secreted the highest quantity of thaxtomin A. In contrast, group I, which is the less virulent group contains strains producing a weak or intermediate amount of thaxtomin A. Even if this production tends to be group-specific, no clear correlation between the quantity of thaxtomin A secreted and the virulence observed was found for the strains under study.

Moreover, among the different pathogenic *Streptomyces* spp. isolated throughout the world, a few reports have indicated that some pathogenic *Streptomyces* are capable of causing CS, while not producing thaxtomin A, usually caused by the absence of the TR region containing the thaxtomin A biosynthetic gene cluster (Wanner, 2004; Cao et al., 2012; Fyans et al., 2016; Lapaz et al., 2017). In this study, we instead identified a pathogenic strain (*S. scabiei* LBUM 1485) that has the entire thaxtomin A biosynthetic cluster but does not produce any detectable amount of thaxtomin A, at least in the standardized *in vitro* assay used here. To our knowledge, this is the second report of a pathogenic *Streptomyces* strain containing the biosynthetic genes encoding thaxtomin A biosynthesis, but which does not produce this phytotoxin (Weisberg et al., 2021).

It has also been previously demonstrated that concanamycin A alone may induce common scab symptoms (Natsume et al., 2017). Therefore, the ability of *S. scabiei* strain LBUM 1485 to cause symptoms on potato tubers may be due to its capacity to produce concanamycin A as it harbors its entire biosynthetic gene cluster, however, this will require further investigation.

In order to understand why LBUM 1485 was not able to produce thaxtomin A, at least under our experimental conditions, the entire thaxtomin A gene cluster was analyzed in detail. Surprisingly, across the 14 *Streptomyces* strains under study, no identifiable polymorphisms within the TR1 region, which may explain variations in virulence phenotypes, were found. The entire regulation system of thaxtomin A production was then compared. No mutation predicted to impact the structure or the functionality of the proteins were found neither in the *txtR* gene directly involved in the transcriptional regulation of the other *txt* genes (Joshi et al., 2007), the *CebR* cellulose utilization regulator, which is known to be the major regulator of virulence in *S. scabies* (Planckaert et al., 2018), nor the CebEFG/MsiK transmembrane transporter involved in the internalization of cellobiose and cellotriose (Jourdan et al., 2016). Moreover, homologs of all of these genes were found in *S. acidiscabies* LBUM 1476 suggesting that the differential production of thaxtomin A between the fourteen strains was not due to differential regulation by these systems. It has already been demonstrated that *bldA* and *bldC* mutants were unable to produce thaxtomin A (Bignell et al., 2014a). Indeed, *bldA* encodes the only leucyl-tRNA that can efficiently translate RNA containing a UUA codon, as found in the *txtR* sequence. A study performed on human tRNA has shown a low frequency of single nucleotide polymorphism (SNP) on the acceptor stem, D-stem, anticodon stem, and T-stem compared to flanking tRNA regions (Thornlow et al., 2018). Thus, the conservation of these regions seems to be particularly important to maintain the tRNA specificity and functionality. In our study, *bldA* was highly conserved among all *S. scabiei* strains, except for *S. scabiei* LBUM 1485, which presents a SNP on the acceptor stem (a G at the position 6 was substituted by a A, which wobbles the G6:C78 link). It was further evidenced that the acceptor stem specifically confers tRNA identity. For example, the G3:U70 or the G1:A82/G2:C81 in *Escherichia coli* Ala-tRNA and Tyr-tRNA, respectively, confer the amino acid accepting specificity (aminoacylation) (Hooper et al., 1972; Celis et al., 1973; Hou and Schimmel, 1988). Considering this observation, this mutation was the best candidate identified in our study to explain the absence of thaxtomin A production by LBUM 1485. Complementary analyses will, however, need to be performed to confirm this observation.

Finally, despite the identification of a mutation in LBUM 1485 potentially explaining the absence of thaxtomin A production by this strain, none of the other comparative analyses performed in this study have been able to highlight with confidence why the fourteen strains display varying virulence. Therefore, using a similar approach as the one used for examining mutations in the thaxtomin A gene cluster, we investigated the potential impact of polymorphisms found on the biosynthetic clusters of all other known virulence determinants involved in the development of potato common scab. Despite the observation of numerous mutations predicted to impact the structure or functionality of the protein encoded, none could explain the virulence pattern observed.

In conclusion, in this study, a high inter-species genomic heterogeneity was found between *S. scabiei* and *S. acidiscabies*, with their respective species-specific pathogenome. Moreover, a high intra-species heterogeneity was also found inside *S. scabiei* with four distinct genomic groups. Despite this group specific pathogenic evolution, no virulence factors were determined to be group specific. Moreover, even among closely related strains belonging to the same group, a distinct pattern of virulence was often observed, suggesting the existence of strains-specific VFs. Indeed, no strong correlation between group affiliation and virulence was found. Similarly, no correlation between virulence and thaxtomin A production was observed, again supporting the existence of yet unknown VFs that are strain-specific. These results highlight the need for sequencing additional scab-causing *Streptomyces* spp., while in parallel characterizing their virulence. Larger-scale analyses will be required to make significant progress toward better understanding the genetic factors involved in virulence/pathogenicity. These progresses will undoubtedly be useful for developing targeted approaches for managing common scab of potato, a disease for which unfortunately no efficient control measure yet exist.

## Data Availability Statement

The datasets presented in this study can be found in online repositories. The names of the repository/repositories and accession number(s) can be found in the article/Supplementary Material.

## Author Contributions

MF designed the research and supervised the work. CH performed the laboratory experiments and data analyses, and wrote the manuscript. AB helped with the bioinformatics analysis, including the design of some associated figures. RS-O helped with laboratory work. AN provided assistance with sequences submission. SL assisted with LC-MS use. JB assisted with the use of the EDGAR platform. All authors were involved in critical reading and reviewing the manuscript.

## Conflict of Interest

The authors declare that the research was conducted in the absence of any commercial or financial relationships that could be construed as a potential conflict of interest.

## Publisher’s Note

All claims expressed in this article are solely those of the authors and do not necessarily represent those of their affiliated organizations, or those of the publisher, the editors and the reviewers. Any product that may be evaluated in this article, or claim that may be made by its manufacturer, is not guaranteed or endorsed by the publisher.

## Footnotes

1
http://provean.jcvi.org/seq_submit.php

2
http://lowelab.ucsc.edu/tRNAscan-SE/index.html

## Abbreviations

AA: amino acid
ANI: average nucleotide identity
*att*: attachment site
BLAST: Basic Local Alignment Search Tool
*cbs*: CebR-binding site
CDSs: coding DNA sequences
CFA-Ile: N-coronafacoyl -L-isoleucine
CIME: *cis-*mobilizable element
CK: cytokinin
CS: common scab
CR: colonization region
*is*DDH: *in silico* DNA–DNA hybridization
GGDC: genome to genome distance calculator
HGT: horizontal gene transfer
IAA: Indole-3-Acetic Acid
ICE: integrative and conjugative elements
LC-MS: liquid chromatography coupled – mass spectrometry
MAFFT: Multiple Alignment using Fast Fourier Transform
PAI: pathogenicity island
PEI: Prince Edward Island
SNP: single nucleotide polymorphism
PROVEAN: Protein Variation Effect Analyzer
TR: toxicogenic region
TSB: tryptic soya broth
VFs: virulence factors
VFDB: Virulence Factor Database.
