# Supplementary material for: Comparative Genomics of Potato Common Scab-Causing Streptomyces spp. Displaying Varying Virulence
Source: Front Microbiol. 2021 Aug 3;12:716522. doi: 10.3389/fmicb.2021.716522 (PMC8369830; doi:10.3389/fmicb.2021.716522)
Supplement: Supplementary file 1 [file Table_1.DOCX]

Supplementary material 1: Genes and/or gene clusters known to be involved in the biosynthesis or regulation of *Streptomyces’* pathogenicity and virulence determinants.

Absence of genes or gene clusters homologue in *S. scabiei* 87-22 or *S. acidiscabies* 84-104 is indicated by “-”.

| **Product** | **Gene/cluster** | **Homologue in *S. scabiei* 87-22** | **Homologue in *S. acidiscabies* 84-104** | **Reference** |
| --- | --- | --- | --- | --- |
| Biosynthesis of thaxtomin A phytotoxin | *txtA* | SCAB_31791 | ON12_RS03045 | Healy et al., 2000 |
|  | *txtB* | SCAB_31781 | ON12_RS03040 | Healy et al., 2000 |
|  | *txtC* | SCAB_31761 | ON12_RS03030 | Healy et al., 2002 |
|  | *txtD* | SCAB_31841 | ON12_RS13200 | Kers et al., 2004b |
|  | *txtE* | SCAB_31831 | ON12_RS13205 | Barry et al., 2012 |
|  | *txtH* | SCAB_31771 | ON12_RS03035 | Bignell et al., 2010 |
| Transcriptional regulator of thaxtomin biosynthetic genes | *txtR* | SCAB_31801 | ON12_RS13210 | Joshi et al., 2007 |
| Global regulators (involved in the biosynthesis of Nec1, thaxtomine A, concanamycin and coronafacoyl) | *bldA* | SCAB_54521 | ON12_RS10960 | Bignell et al., 2014 |
|  | *bldC* | SCAB_47901 | ON12_RS09880 |  |
|  | *bldD* | SCAB_75171 | ON12_RS35955 |  |
|  | *bldG* | SCAB_40861 | ON12_RS37750 |  |
|  | *bldH* | SCAB_57831 | ON12_RS01295 |  |
| CebEFG/Msik transporter | *cebE* | SCAB_57751 | ON12_RS01325 | Jourdan et al., 2016 |
|  | *cebF* | SCAB_57741 | ON12_RS01330 |  |
|  | *cebG* | SCAB_57731 | ON12_RS01335 |  |
|  | *msiK* | SCAB_570161 | ON12_RS27170 |  |
| Cellobiose utilization regulator | *cebR* | SCAB_57761 | ON12_RS01320 | Francis et al., 2015 |
| Concanamycin A | Biosynthesis gene cluster of *S. neyagawaensis* ATCC27449 | From SCAB_83851 to SCAB_84101 | - | Haydock et al., 2005 |
| N-coronafacoyl-l-isoleucine (CFA) | CFA biosynthetic and regulatory genes | From SCAB_79581 to SCAB_79721 | - | Bown et al., 2017 |
| Borrelidin | Biosynthesis cluster of *S. parvulus* Tu4055 | - | - | Olano et al., 2004 |
| Desmethylmensacarcin | Mensacarcin biosynthetic gene cluster from *S. bottropensis* | - | From ON12_RS17810 to ON12_RS17925 | Yan et al., 2012 |
| FD-891 | Biosynthetic cluster of *S. graminofaciens* A‐8890 | - | - | Kudo et al., 2010 |
| Auxin (indole-3-acetic acid) | Indole-3-acetamide pathway (*iaaH* and *iaaM*) | SCAB_75501and SCAB_75511 | ON12_RS36085 and ON12_RS36090 | Hsu, 2010 |
| Ethylene | Ethylene-forming enzyme gene (*efe*) | SCAB_86241 and SCAB_51351 | ON12_RS35420 | Tomihama et al., 2016 |
| Cytokinins | *fas* operon | - | - | Kers et al., 2004a |
| Nec1 | *nec1* | SCAB_77602 | ON12_RS51950 | Bukhalid et al., 1998 |
| TomA | *tomA* | SCAB_77321 | ON12_RS19355 | Kers et al., 2004a |
| Scabin | Scabin gene | SCAB_27771 | ON12_RS00390 | Lyons et al., 2016 |
| Suberinase | *sub1* | SCAB_78931 | - | Komeil et al., 2013 |

Barry, S. M., Kers, J. A., Johnson, E. G., Song, L., Aston, P. R., Patel, B., et al. (2012). Cytochrome P450–catalyzed L-tryptophan nitration in thaxtomin phytotoxin biosynthesis. Nat. Chem. Biol. 8, 814–816.

Bignell, D. R. D., Francis, I. M., Fyans, J. K., and Loria, R. (2014). Thaxtomin A production and virulence are controlled by several *bld* gene global regulators in *Streptomyces* *scabies*. Mol. Plant Microbe Interact. 27, 875–885. doi:10.1094/MPMI-02-14-0037-R.

Bignell, D. R. D., Huguet-Tapia, J. C., Joshi, M. V., Pettis, G. S., and Loria, R. (2010). What does it take to be a plant pathogen: genomic insights from *Streptomyces* species. Antonie van Leeuwenhoek 98, 179–194. doi:10.1007/s10482-010-9429-1.

Bown, L., Li, Y., Berrué, F., Verhoeven, J. T. P., Dufour, S. C., and Bignell, D. R. D. (2017). Coronafacoyl phytotoxin biosynthesis and evolution in the common scab pathogen *Streptomyces* *scabiei*. Applied and Environmental Microbiology 83, e01169-17. doi:10.1128/AEM.01169-17.

Bukhalid, R. A., Chung, S. Y., and Loria, R. (1998). *nec1*, a gene conferring a necrogenic phenotype, is conserved in plant-pathogenic *Streptomyces* spp. and linked to a transposase pseudogene. Mol. Plant Microbe Interact. 11, 960–967. doi:10.1094/MPMI.1998.11.10.960.

Francis, I. M., Jourdan, S., Fanara, S., Loria, R., and Rigali, S. (2015). The cellobiose sensor CebR is the gatekeeper of *Streptomyces* *scabies* pathogenicity. MBio 6. doi:10.1128/mBio.02018-14.

Haydock, S. F., Appleyard, A. N., Mironenko, T., Lester, J., Scott, N., and Leadlay, P. F. (2005). Organization of the biosynthetic gene cluster for the macrolide concanamycin A in *Streptomyces* *neyagawaensis* ATCC 27449. Microbiology 151, 3161–3169.

Healy, F. G., Krasnoff, S. B., Wach, M., Gibson, D. M., and Loria, R. (2002). Involvement of a cytochrome P450 monooxygenase in thaxtomin A biosynthesis by *Streptomyces* *acidiscabies*. J. Bacteriol. Res. 184, 2019–2029. doi:10.1128/JB.184.7.2019-2029.2002.

Healy, F. G., Wach, M., Krasnoff, S. B., Gibson, D. M., and Loria, R. (2000). The *txtAB* genes of the plant pathogen *Streptomyces* *acidiscabies* encode a peptide synthetase required for phytotoxin thaxtomin A production and pathogenicity. Mol. Microbiol. 38, 794–804. doi:10.1046/j.1365-2958.2000.02170.x.

Hsu, S.-Y. (2010). IAA production by *Streptomyces scabies* and its role in plant microbe interaction. Available at: https://hdl.handle.net/1813/17227.

Joshi, M. V., Bignell, D. R. D., Johnson, E. G., Sparks, J. P., Gibson, D. M., and Loria, R. (2007). The AraC/XylS regulator TxtR modulates thaxtomin biosynthesis and virulence in *Streptomyces scabies*. Mol. Microbiol. 66, 633–642. doi:10.1111/j.1365-2958.2007.05942.x.

Jourdan, S., Francis, I. M., Kim, M. J., Salazar, J. J. C., Planckaert, S., Frère, J.-M., et al. (2016). The CebE/MsiK transporter is a doorway to the cello-oligosaccharide-mediated induction of *Streptomyces scabies* pathogenicity. Sci. Rep. 6. doi:10.1038/srep27144.

Kers, J. A., Cameron, K. D., Joshi, M. V., Bukhalid, R. A., Morello, J. E., Wach, M. J., et al. (2004a). A large, mobile pathogenicity island confers plant pathogenicity on *Streptomyces* species: *S. turgidiscabies* pathogenicity island. Mol. Microbiol. 55, 1025–1033. doi:10.1111/j.1365-2958.2004.04461.x.

Kers, J. A., Wach, M. J., Krasnoff, S. B., Widom, J., Cameron, K. D., Bukhalid, R. A., et al. (2004b). Nitration of a peptide phytotoxin by bacterial nitric oxide synthase. Nature 429, 79–82.

Komeil, D., Simao-Beaunoir, A.-M., and Beaulieu, C. (2013). Detection of potential suberinase-encoding genes in *Streptomyces scabiei* strains and other actinobacteria. Can. J. Microbiol. 59, 294–303. doi:10.1139/cjm-2012-0741.

Kudo, F., Motegi, A., Mizoue, K., and Eguchi, T. (2010). Cloning and characterization of the biosynthetic gene cluster of 16-membered macrolide antibiotic FD-891: Involvement of a dual functional cytochrome P450 monooxygenase catalyzing epoxidation and hydroxylation. Chem. Eur. J. of Chem. Bio. 11, 1574–1582. doi:10.1002/cbic.201000214.

Lyons, B., Ravulapalli, R., Lanoue, J., Lugo, M. R., Dutta, D., Carlin, S., et al. (2016). Scabin, a novel DNA-acting ADP-ribosyltransferase from *Streptomyces scabies*. J. Biol. Chem. 291, 11198–11215. doi:10.1074/jbc.M115.707653.

Olano, C., Moss, S. J., Braña, A. F., Sheridan, R. M., Math, V., Weston, A. J., et al. (2004). Biosynthesis of the angiogenesis inhibitor borrelidin by *Streptomyces parvulus* Tü4055: insights into nitrile formation. Mol. Microbiol. 52, 1745–1756.

Tomihama, T., Nishi, Y., Sakai, M., Ikenaga, M., Okubo, T., and Ikeda, S. (2016). Draft genome sequences of *Streptomyces scabiei* S58, *Streptomyces turgidiscabies* T45, and *Streptomyces acidiscabies* a10, the pathogens of potato common scab, isolated in Japan. Genome Announc., 4.

Yan, X., Probst, K., Linnenbrink, A., Arnold, M., Paululat, T., Zeeck, A., et al. (2012). Cloning and heterologous expression of three type II PKS gene clusters from *Streptomyces* *bottropensis*. Chem. Bio. Chem 13, 224–230.
