# Supplementary material for: Comparative Genomics of Potato Common Scab-Causing Streptomyces spp. Displaying Varying Virulence
Source: Front Microbiol. 2021 Aug 3;12:716522. doi: 10.3389/fmicb.2021.716522 (PMC8369830; doi:10.3389/fmicb.2021.716522)
Supplement: Supplementary file 4 [file Table_4.DOCX]

Supplementary material 4: Impact of mutations on the predicted secondary structure of *bldA*, the only a Leu-tRNA^UUA^ in phytopathogenic *S. scabiei* genomes.

The predicted secondary structure of Leu tRNA^UUA^ was determined using tRNAscan-SE (http://lowelab.ucsc.edu/tRNAscan-SE/index.html).

**
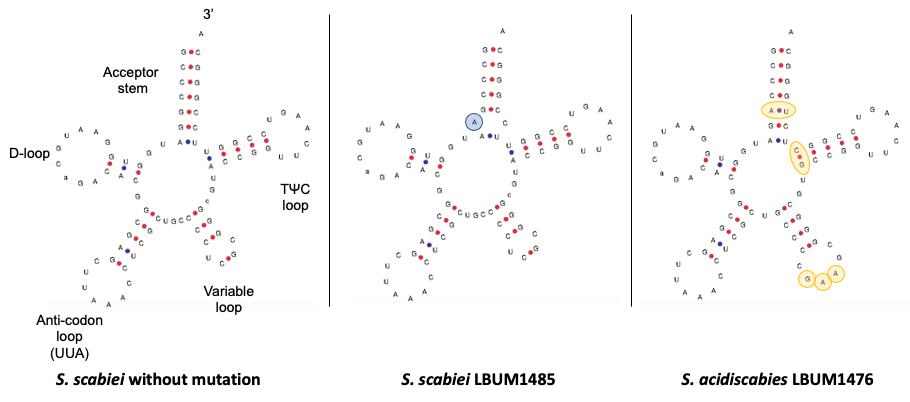
**
