# Supplementary material for: Comparative Genomics of Potato Common Scab-Causing Streptomyces spp. Displaying Varying Virulence
Source: Front Microbiol. 2021 Aug 3;12:716522. doi: 10.3389/fmicb.2021.716522 (PMC8369830; doi:10.3389/fmicb.2021.716522)
Supplement: Supplementary file 5 [file Table_5.DOCX]

Supplementary material 5: Phylogenetic tree of the concatenated protein sequences of all virulence determinants involved in the CS disease.

The neighbor-Joining tree was based on the MAFFT alignment of the 37 protein sequences concatenated containing mutations presented in supplementary material 3. Branches are presented as a cladogram for improved clarity. The bootstrap values based on 1,000 replicates are displayed next to the branches.
